# Body Composition and Adiposity in Children with Down Syndrome Compared to Typically Developing Children: The Association with Their Motor Performance

**DOI:** 10.3390/children12101298

**Published:** 2025-09-25

**Authors:** Dhoha W. Binsaddiq, Alaa I. Ibrahim, Turki S. Abualait

**Affiliations:** 1 Department of Physical Therapy, Saud Al Babtain Cardiac Center, Dammam 32245, Saudi Arabia; 2 Department of Physical Therapy, Imam Abdulrahman Bin Faisal University, Dammam 31451, Saudi Arabia; aiibrahim@iau.edu.sa (A.I.I.); tsabualait@iau.edu.sa (T.S.A.)

**Keywords:** Down syndrome, body composition, adiposity, physical activity, balance, muscle strength

## Abstract

**Highlights:**

**What are the main findings?**

- Children with Down syndrome (DS) exhibited significantly greater adiposity and reduced motor performance outcomes compared to their typically developing (TD) counterparts.
- In children with DS, increased fat mass demonstrated strong negative correlations with balance, aerobic fitness, and physical activity levels.

**What are the implications of the main findings?**

- These findings highlight a negative feedback loop, wherein excess adiposity in children with DS contributes to motor impairments, which in turn further limit physical activity and functional capacity.
- The results underscore the need for early, multidimensional interventions targeting the promotion of physical activity and weight management to mitigate health risks and improve developmental outcomes in this population, particularly in regions with higher prevalence rates such as Saudi Arabia.

**Abstract:**

**Background/Objectives:** Children with DS are at an increased risk of obesity and impaired motor performance. This study aimed to compare body composition and motor performance in children with DS and typically developing (TD) peers and to explore associations between adiposity and motor function. **Methods:** A cross-sectional study was conducted with 42 children aged 6–12 years (23 DS, 19 TD). Body composition was assessed using bioelectrical impedance analysis. Postural stability was evaluated with the Pediatric Balance Scale; hand grip strength with hand dynamometry; physical activity with the PAQ-C; and aerobic endurance with the YMCA 3 min step test. **Results**: Children with DS exhibited significantly higher adiposity and lower motor performance than their TD peers. In the DS group, body fat mass was negatively correlated with physical activity (r = –0.499, *p* = 0.018), balance (r = –0.684, *p* < 0.001), and aerobic endurance (r = –0.389, *p* < 0.073). Regression analysis identified physical activity and fitness level as significant predictors of BMI in children with DS (R^2^ = 0.825). **Conclusions**: Children with DS exhibit higher adiposity and inferior motor performance compared to their TD peers, with strong associations between adiposity and reduced physical activity, balance, and aerobic endurance. These findings underscore the importance of early targeted interventions to improve health outcomes in children with DS, particularly in regions like Saudi Arabia, where prevalence is high.

## 1. Introduction

Down syndrome (DS) is a genetic disorder characterized by a range of clinical features that arise from the total or partial manifestation of an additional chromosome 21, further referred to as trisomy 21 [1]. The estimated global prevalence of DS is 0.1% of every live birth, making it the most prevalent form of autosomal aneuploidy that causes intellectual disabilities [2,3]. Higher prevalence rates are observed in Saudi Arabia, where 1 in 554 live births are affected by DS [4]. Moreover, recent estimations recorded a prevalence of 6.6 per 10,000 children [5].

Children with DS display a higher incidence of obesity compared to those with other forms of intellectual disability [6,7] and exhibit two to three times higher obesity rates than their neurotypical peers [8]. Although obesity varies depending on age, data suggest that more than 60% of children with DS are overweight, as defined by a body mass index (BMI) surpassing the 85th percentile [8]. Moreover, this figure is anticipated to surge to 70–80% in adulthood [9]. The escalated rates of obesity in children with DS are partially attributed to their gravitation toward sedentary behavior (SB) and lack of engagement in moderate-to-vigorous physical activity [10]. Children with obesity tend to have lower fitness and aerobic endurance levels relative to their peers with lower body fat percentages [11]. However, whether this correlation applies to children with DS remains uncertain. That said, a deficit in aerobic endurance levels exists in individuals with DS compared to their healthy counterparts and age-matched peers with other intellectual disabilities [12,13]. These deficits are primarily observed in the lower limb muscles, as resistance training has been found to improve upper limb endurance with minute changes to the lower extremity in the targeted population [14].

Motor dysfunction in children with DS may vary, with some children experiencing deficiencies in muscle strength, aerobic endurance, and postural stability [15,16,17]. There are observable disparities in muscle strength between children with DS and TD children, with the former displaying lower muscle strength than their TD peers [18].

It is well noted that PA levels and BMI significantly predict muscle strength in children with DS and their TD peers [18]. Consequently, children with DS who take preference in SB tend to have higher BMI levels and lower mean muscle strength values than physically active TD children [18,19]. According to the guidelines set forth by the World Health Organization (WHO), PA is recommended for youth between the ages of 5 and 17, with a minimum of 60 min per day of moderate-to-vigorous-intensity PA, primarily aerobic [20]. Nevertheless, the majority of children with DS do not meet these guidelines [21].

The ability to maintain postural stability is a fundamental aspect of motor development and is crucial to executing proficient movements [2]. Children with DS typically exhibit significantly impaired static and dynamic balance abilities compared to TD children [2]. These impairments are primarily observed in novel, challenging conditions [22]. Postural stability in the medial–lateral and anterior–posterior stability indices, as well as the overall stability index, is significantly impaired in children with DS compared to healthy individuals [23].

The relationship between motor performance and PA has been articulated within the developmental model of motor competence [24]. This framework illustrates a reciprocal and reinforcing cycle: children who exhibit higher motor competence are more likely to engage in increased PA, which subsequently contributes to healthier body composition and the further advancement of motor skills. Conversely, children with lower motor skills may be less active, leading to increased adiposity and diminished physical fitness. This cycle is particularly salient for children with DS, as impairments in motor skills can exacerbate low levels of PA and negatively impact body composition trajectories.

Although the interplay between body composition, adiposity, motor performance, and PA in children with DS is widely acknowledged, studies examining these combined associations remain scarce, particularly in Middle Eastern populations where cultural, environmental, and dietary factors may exert significant influence [25]. In Saudi Arabia, the prevalence of DS is higher than global averages, with obesity rates showing a concerning upward trend; however, evidence on motor performance in this group remains limited [3,4,26]. Addressing this gap, the present study compared the body composition between children with DS and their TD peers, and examined associations with motor performance indicators—postural stability, hand grip strength, and aerobic endurance. Based on this objective, we hypothesized that children with DS would demonstrate greater adiposity and altered body composition compared to their TD peers, with these differences being associated with poorer motor performance outcomes and lower levels of PA.

## 2. Materials and Methods

### 2.1. Study Design and Participants

This cross-sectional observational study comprised 42 children between the ages of 6 and 12 years, including 23 with Down syndrome (DS) and 19 typically developing (TD) children, selected through a non-probabilistic sampling method. While this methodology facilitated access to the intended participant population, it may have also introduced potential selection bias, indicating that the findings may not wholly represent the variability associated with diverse socioeconomic or environmental contexts. This limitation is acknowledged and should be carefully considered in the interpretation of the results. Children with DS were recruited from AL Qimma Day center, while TD peers were recruited from Al Fursan International schools. Ethical approval was granted by the Institutional Review Board of Imam Abdulrahman bin Faisal University (IRB-2023-PTDS-001). Informed consent was obtained from all legal guardians.

### 2.2. Inclusion and Exclusion Criteria

Children were included if they were aged 6–12 years and were capable of following instructions. For the DS group, a confirmed diagnosis of trisomy 21 was required. Exclusion criteria included congenital heart disease, uncorrected sensory impairments, and orthopedic or neurological conditions affecting mobility.

### 2.3. Sample Size

Sample size was calculated using G*Power 3.1 software based on expected effect sizes from previous studies [27], with α = 0.05 and power = 0.95. A minimum of 19 participants per group was deemed adequate.

### 2.4. Body Composition and Adiposity

Bioelectrical impedance analysis (BIA) using an X-scan Plus (Jawon Medical Co., Daejeon, Korea) was used to assess body composition in participants due to its practicality and reliability (ICC = 0.999; sensitivity = 0.78) [28]. The test was conducted in a controlled environment (20 °C), following manufacturer guidelines. Participants rested for 10 min beforehand to stabilize body fluids and provided basic demographic data for input. During the test, children stood barefoot on the device in a standardized posture, with precautions taken to avoid interference (e.g., removing conductive items, using a non-conductive mat, wiping hands/feet, and avoiding food or strenuous activity before testing). The measurement lasted 2–3 min, and the results were printed afterward. Any invalid readings due to movement were repeated.

### 2.5. Physical Activity

Physical activity (PA) was measured using the validated Arabic version of the Physical Activity Questionnaire for Older Children (PAQ-C), suitable for children aged 8–14. The tool demonstrates good reliability (Cronbach’s α = 0.74; test–retest r = 0.75–0.82) and validity in school-aged Saudi children [26,29]. PAQ-C is a self-administered, 7-day recall questionnaire with nine items rated on a 5-point scale, covering activity in physical education, recess, after-school activities, and sports. It was administered (1:1) by a trained researcher in the Exercise Physiology Laboratory at Imam Abdulrahman bin Faisal University. To ensure standardized administration and minimize recall bias, a standardized script was administered, questions were read aloud, and clarifications were provided as needed without influencing responses. Guardians were instructed to recall their children’s activities from the previous 7 days, and visual aids were used when appropriate to enhance comprehension. All participants received identical instructions and were tested in equal conditions at a similar time of day. Subsequently, the mean score was calculated, with 1 indicating low and 5 indicating high PA.

### 2.6. Postural Stability

Postural stability was assessed using the Pediatric Balance Scale (PBS), a valid and reliable tool (ICC = 0.998) for children with or without motor or neurological impairments [30]. Additionally, it displays convergent validity with established scales such as the Gross Motor Function Measure (GMFM) and the Pediatric Evaluation of Disability Inventory (PEDI) [31] The PBS consists of 14 tasks assessing static and dynamic balance, scored 0–4 per task, with a total possible score of 56. Tests were conducted in a safe, obstacle-free environment, with clear instructions, pilot testing, and demonstrations provided to ensure participant understanding.

### 2.7. Hand Grip Strength

Hand grip strength was assessed using a JAMAR^®^ Hydraulic Hand-Held Dynamometer (Hand Evaluation Kit, Patterson Medical Holdings, Inc., Mississauga, Ontario, Canada) to measure grip strength (ICC = 0.91–0.93) and key pinch strength (ICC > 0.90), both recognized as valid and reliable tools [32]. Participants, seated with correct posture, completed three maximum-effort grip trials on each hand, beginning with the dominant side. The mean of the three trials was calculated as the final grip strength.

### 2.8. Aerobic Endurance

Aerobic endurance was assessed using the YMCA 3 min step test, a valid and reliable measure of aerobic endurance (ICC = 0.90) and a standardized field test validated for use in children aged 6–12 years [33]. The test has also demonstrated good construct validity and intra-rater reliability in children aged 7–11 years [34]. Participants stepped on a 12-inch platform at 96 steps per minute for three minutes, with perceived exertion monitored throughout. Post-exercise heart rate was recorded and compared to fitness category charts, where lower rates indicated better aerobic endurance. The test was conducted safely in a controlled environment, with pre- and post-test vital signs, loose clothing, and precautions for fatigue or distress, ensuring participants could stop if needed.

### 2.9. Statistical Analysis

Data were analyzed using SPSS version 27. Normality was assessed using the Shapiro–Wilk test. Independent *t*-tests or Mann–Whitney U tests were applied for between-group comparisons. Pearson or Spearman correlation coefficients were calculated to examine associations between variables. Stepwise multiple linear regression was used to identify predictors of adiposity. A significance level of *p* < 0.05 was used.

## 3. Results

### 3.1. Demographic and Anthropometric Characteristics

A total of 43 participants were included in this study, comprising 22 males (51%) and 21 females (49%). The mean age of the participants was 9.32 ± 1.34 years (range: 8–12 years). Among them, 21 children were TD, while 22 were diagnosed with DS. The TD sample consisted of 52.4% males and 47.6% females, with a mean age of 8.90 ± 1.14 years. Their mean height and weight were 125.43 ± 6.01 cm and 25.52 ± 4.69 kg, respectively. The DS male–female ratio was equal (50%), with a mean age of 9.73 ± 1.42 years (range: 8–12). The DS children’s average weight and height were 38.17 ± 12.18 kg and 127.50 ± 10.17 cm, respectively.

### 3.2. Comparing Body Composition Parameters

Table 1 compared body composition parameters between TD children and children with DS Children with DS exhibited a significantly higher weight (38.168 ± 12.17 vs. 25.519 + 4.693 kg, *p* = 0.001), higher BMI (23.1864 ± 4.45168 vs. 16.1048 ± 1.61601 kg/m^2^, *p* = 0.001), higher body fat mass (10.23 ± 5.62 vs. 3.48 ± 1.72 kg, *p* = 0.001), and percentage of body fat (26 vs. 14%, *p* = 0.001) compared to TD children, indicating greater adiposity. Despite an increased lean body mass in DS children (+5.75 kg, *p* = 0.006), their basal metabolic rate was significantly higher (+60.30 kcal/day, *p* = 0.031), likely due to differences in body composition.

### 3.3. Comparing Postural Stability Characteristics

Table 2 compares postural stability characteristics between TD children and children with DS. Postural stability measures were consistently lower in children with DS compared to TD children, with significant reductions in total balance scores (45.77 ± 5.62 vs. 55.71 ± 0.9, respectively, *p* = 0.001) and critical tasks such as standing with one foot in front (2.18 ± 0.66 vs. 3.95 ± 0.22, respectively, *p* = 0.001) and placing alternate feet on a stool (2.91 ± 0.68 vs. 4.00, respectively, *p* = 0.001).

### 3.4. Comparing Muscle Strength, Physical Activity, and Endurance Characteristics

Table 3 compares hand grip strength, physical activity, and aerobic endurance characteristics between TD children and children with DS. Key pinch strength was significantly lower in children with DS compared to TD children (2.95 ± 0.84 vs. 3.9 ± 0.7, respectively, *p* = 0.001). Similarly, the PA composite scores were substantially lower in children with DS (1.77 ± 0.75 vs. 4.05 ± 0.74, respectively, *p* = 0.001), and aerobic endurance was markedly reduced, as evidenced by a significantly higher post-exercise heart rate (124.64 ± 11.16 vs. 91.43 ± 12.95 bpm, respectively, *p* = 0.001).

### 3.5. Association Between Body Composition Parameters and Study Outcome Measures

Table 4 presents bivariate correlation coefficients for body composition variables with demographic characteristics, postural stability, physical activity, hand grip strength, and aerobic endurance in TD children. When focusing on the motor performance correlations, significant positive correlations were found between BMI and PA composite scores (r = 0.485, *p* = 0.026), hand grip strength (r = 0.578, *p* = 0.006), and key pinch strength (r = 0.557, *p* = 0.009), indicating that a higher body mass index is associated with increased physical activity and hand grip strength. Similarly, lean body mass was positively correlated with PA composite scores (r = 0.729, *p* < 0.001), hand grip strength (r = 0.777, *p* < 0.001), and key pinch strength (r = 0.627, *p* = 0.002), indicating that a higher fat-free mass is associated with increased physical activity and hand grip strength. It is essential to mention that no association was recorded between postural stability and aerobic endurance or any of the body composition parameters.

Table 5 presents bivariate correlation coefficients for body composition variables with demographic characteristics, postural stability, physical activity, hand grip strength, and aerobic endurance in children with DS. When focusing on the motor performance correlations, the body mass index (BMI), lean body mass (LBM), and body fat mass (MBF) had similar positive correlations with hand grip strength (BMI: r = 0.688, *p* < 0.001; LBM: r = 0.891, *p* < 0.001; MBF: r = 0.810, *p* < 0.001), key pinch strength (BMI: r = 0.436, *p* = 0.043; LBM: r = 0.587, *p* = 0.004; MBF: r = 0.468, *p* = 0.028), and the 1 min post-exercise HR (BMI: r = 0.662, *p* = 0.001; LBM: r = 0.658, *p* = 0.001; MBF: r = 0.673, *p* = 0.001), indicating that a higher body mass index, fat-free mass, and body fat mass are associated with increased hand grip strength and post-exercise HR but with decreased aerobic endurance.

On the other hand, BMI, LBM, and MBF had negative correlations with postural stability (BMI: r = −0.697, *p* < 0.001; LBM: r = −0.827, *p* < 0.001; MBF: r = −0.684, *p* < 0.001), physical activity (BMI: r = −0.694, *p* < 0.001; LBM: r = −0.536, *p* = 0.010; MBF: r = −0.449, *p* < 0.018), and the fitness category (BMI: r = −0.714, *p* = 0.001; LBM: r = −0.476, *p* = 0.025; MBF: r = −0.389, *p* < 0.073), indicating that a higher body mass index, fat-free mass, and body fat mass are associated with a reduction in postural stability, physical activity, and aerobic endurance.

Table 6 presents multiple linear regression models identifying the significant body composition predictors in TD children. No significant predictors were detected for the BMI. LBM was significantly predicted by gender (β = 0.471, *p* = 0.004) and height (β = 0.768, *p* = 0.003), while MBF was predicted only by gender (β = −0.727, *p* = 0.003).

Table 7 presents multiple linear regression models identifying the significant body composition predictors in children with DS. BMI was significantly predicted by PA composite score (β = −0.359, *p* = 0.029) and fitness category (β = −0.517, *p* = 0.029). LBM was significantly predicted by height (β = 0.585, *p* = 0.039) and hand grip strength (β = 0.429, *p* = 0.049). MBF had no significant predictors.

## 4. Discussion

This study compared body composition and adiposity between children with DS and their TD peers, and examined their association with motor performance and physical activity. Children with DS had a significantly higher weight, BMI, total fat mass, and body fat percentage, as well as greater central (trunk) fat deposition, compared to their TD peers. These findings align with existing evidence that youth with DS are at higher risk of obesity than the general pediatric population and those with other intellectual disabilities [6]. Multiple physiological and behavioral factors are likely to contribute to the increased rates of adiposity observed in children with Down syndrome (DS). These factors encompass a decreased resting metabolic rate, endocrine abnormalities such as hypothyroidism, and variations in leptin levels [35]. Additionally, children with DS exhibit lower levels of PA compared to their TD peers, a pattern that emerges even in early childhood [36]. Together, these factors promote positive energy balance and fat accumulation. The pattern of increased trunk fat, consistent with previous findings, may indicate elevated cardiometabolic risk in this population [37].

Alongside their altered body composition, children with Down syndrome (DS) presented significantly poorer motor performance when compared to their typically developing (TD) peers. They exhibited reduced balance and postural stability, with lower total balance scores and difficulty performing tasks requiring advanced coordination (e.g., tandem stance, alternate step-ups). These deficits are consistent with the neuromuscular characteristics of DS—hypotonia, ligamentous laxity, reduced strength, and impaired postural control—which contribute to delayed motor development and less efficient movement patterns [16]. These findings confirm that balance challenges persist into school age. Furthermore, children with DS demonstrated significantly lower physical activity (PA) and aerobic endurance levels, reflected by lower composite PA scores, indicating reduced participation and proficiency in age-appropriate PA.

Following the 3 min step test, children with Down syndrome (DS) exhibited higher one-minute post-exercise heart rates than their typically developing (TD) peers, indicating slower cardiovascular recovery and lower cardiorespiratory fitness. This is consistent with evidence showing that youth with DS engage less in moderate-to-vigorous physical activity and have a lower aerobic capacity [38]. Contributing factors include motor impairments and muscle weakness, which make physical activity (PA) more difficult and less enjoyable, as well as overprotective caregiving and limited inclusive opportunities [14]. Physiologically, a blunted maximal heart rate and lower peak oxygen uptake in DS [39] may explain this elevated post-exercise heart rate and reduced aerobic endurance. Together, these findings underscore a vicious cycle in which reduced fitness and PA exacerbate the higher adiposity and motor difficulties already present in children with DS.

Comparable findings have emerged from studies conducted in sociocultural contexts similar to that of Saudi Arabia, indicating that lifestyle behaviors, cultural norms, and environmental factors play a significant role in shaping children’s physical activity levels and body composition. For instance, [25] reported that children with Down syndrome (DS) in Saudi Arabia exhibit significantly lower levels of physical activity compared to their typically developing peers. This disparity is partially attributable to a lack of inclusive school-based activity programs and limited access to recreational facilities. Similar patterns observed in Middle Eastern and South Asian populations suggest that cultural expectations, dietary practices, and socioeconomic constraints may collectively exacerbate sedentary behavior and increase obesity risk among children with DS [40].

These observations reinforce the concept of a “vicious cycle,” wherein increased adiposity contributes to decreased participation in physical activity, leading to motor decline and further weight gain. However, this cycle is not solely biologically driven; it is also influenced by environmental and sociocultural barriers, including parental overprotection, insufficient opportunities for adapted sports, and limited awareness of the importance of structured physical activity. Effectively breaking this cycle necessitates targeted multidisciplinary interventions that integrate adapted physical activity programs, individualized nutritional counseling, and education for both parents and families. Evidence indicates that early, structured physical activity interventions tailored for children with DS can enhance fitness, motor proficiency, and levels of participation, while dietary counseling can assist in managing caloric intake and reducing the risk of obesity [28,41].

Therefore, the collaboration of physical therapists, nutritionists, educators, and caregivers is essential in designing holistic, culturally sensitive interventions. By simultaneously addressing biological predispositions and sociocultural barriers, such programs may effectively mitigate the long-term health risks associated with obesity and motor decline within this population.

The relationship between adiposity and motor performance differed markedly between groups. In typically developing (TD) children, modest positive correlations were found between BMI and specific motor outcomes, specifically measures of hand grip muscle strength coupled with PA composite scores. However, BMI was not significantly associated with postural stability, fitness, or post-exercise heart rate in the TD group. Although higher adiposity is generally detrimental to motor function, this finding likely reflects standard growth patterns, as older and more mature children tend to have higher BMIs due to increased height and muscle mass, which also contribute to greater strength and PA [32,42]. Importantly, this does not suggest that excess fat is beneficial; obesity in TD youth is associated with impaired motor skills and balance [43,44]. The absence of a negative association between BMI and postural stability or aerobic endurance in the TD group likely reflects their generally healthy weight status and the dominant influence of age and developmental stage on performance. In this cohort, body composition did not appear to limit motor performance among TD children once normal growth-related differences were accounted for.

In children with DS, adiposity and body composition measures (BMI, fat mass, lean mass) showed moderate-to-strong negative correlations with key motor outcomes and physical activity levels. A higher BMI and fat mass were linked to lower physical activity (PA) scores, poorer aerobic endurance, and impaired postural stability. Excess weight likely exacerbates biomechanical and neuromuscular challenges inherent to DS, such as hypotonia, joint laxity, and coordination difficulties, leading to greater instability and fatigue during movement [39,44]. Increased adiposity was also associated with reduced daily activity, creating a vicious cycle of low activity and weight gain [45]. Interestingly, body size correlated positively with hand grip strength and post-exercise heart rate, reflecting greater absolute force in children with higher BMIs but not translating into improved balance or aerobic fitness. Although absolute grip strength was measured in this study, it is important to note that relative grip strength (i.e., grip strength normalized to body mass) provides more functional insight, especially in children with DS who tend to have higher adiposity. Prior research has shown that despite their higher body weight, children with DS often display reduced relative strength, which can limit functional performance and participation in daily activities [28,46]. Moreover, elevated post-exercise heart rates may indicate greater cardiovascular strain and slower recovery, possibly due to obesity-related autonomic dysfunction [47]. Overall, excess weight worsens motor proficiency and fitness in children with DS, amplifying their underlying motor deficits.

The regression analysis highlighted distinct predictors of body composition in children with Down syndrome (DS) versus typically developing (TD) children. In the TD group, biological factors—sex and height—were the primary predictors of BMI and body composition, consistent with normal growth and pubertal development trends [48]. Males exhibited lower fat mass and higher lean mass, while taller children had greater lean tissue. Importantly, motor performance, physical activity (PA), and fitness variables did not significantly predict BMI in TD children, potentially reflecting a relatively healthy, moderate activity level in the sample and the dominant influence of genetic and maturational factors on BMI at this developmental stage [49]. Conversely, in children with DS, modifiable lifestyle factors played a critical role: lower PA composite scores and poorer aerobic endurance independently predicted a higher BMI, suggesting a more substantial impact of behavior and functional status on body composition in this population. This supports the prior literature identifying physical inactivity as a key contributor to obesity in DS [6,50]. The DS group’s lower baseline metabolic rate and hypotonia may heighten sensitivity to variations in activity, magnifying their effect on energy balance and adiposity. Furthermore, hand grip strength and height significantly predicted lean body mass in DS children, indicating variability in muscle development potentially influenced by PA levels, early therapy, and genetics. Unlike the TD group, sex did not significantly influence body composition in DS, possibly due to a smaller sample size, limited pubertal variation, or trisomy 21′s overriding effect on growth patterns [6,51].

Collectively, these findings depict a complex interplay in DS between greater adiposity and reduced motor proficiency and fitness. Physiological factors such as hypotonia, muscle weakness, and neurodevelopmental anomalies delay motor milestones and impair balance [16]. These impairments reduce participation in vigorous physical activities, fostering sedentary behaviors that promote fat accumulation—exacerbated by lower basal energy expenditure and possible dietary challenges [29,52]. Excess adiposity, in turn, intensifies motor difficulties by increasing biomechanical load and fatigue, potentially accompanied by psychosocial barriers like diminished confidence, creating a vicious cycle [8]. The observed moderate-to-strong negative correlations between adiposity and motor performance in DS children empirically underscore this cycle. Without intervention, these intertwined deficits may compound through adolescence and adulthood, heightening health risks. Clinically, these results advocate for early, integrated interventions targeting motor skill development, physical activity promotion, and weight management in children with DS. Physical therapy aimed at enhancing core strength, balance, and coordination may empower greater participation in PA, mitigating disparities in body composition and motor outcomes. Future longitudinal studies are warranted to elucidate causal pathways and evaluate the efficacy of combined therapeutic and lifestyle approaches. In sum, addressing fitness, motor function, and adiposity concurrently offers the best prospects for breaking the negative feedback loop inherent in DS and supporting optimal developmental and health trajectories [20].

## 5. Limitations

The small sample size (n = 42), cross-sectional design, and non-probabilistic recruitment may introduce selection bias and limit generalizability and causal inference. Recruitment from a single geographic area further reduces external validity, as unmeasured socioeconomic and environmental factors could have influenced physical activity and body composition outcomes. Although validated tools were used, methodological constraints exist: the PAQ-C relies on self-report, bioelectrical impedance analysis (BIA) is less precise than gold-standard methods like DEXA, and motor performance assessments (grip strength, YMCA 3-min step test) may have been affected by hypotonia, compliance variability, or ceiling effects. Several potential confounders, including diet, socioeconomic status, comorbidities, and hormonal factors, were not assessed, and the limited sample precluded stratified analyses by age or pubertal stage.

## 6. Conclusions

Children with Down syndrome exhibit significantly greater adiposity and impaired motor performance compared to typically developing peers. Adiposity in this population is strongly associated with reduced physical activity, postural instability, and lower aerobic endurance. Modifiable factors such as physical activity, hand grip strength, and aerobic endurance were identified as significant predictors of fat mass in children with DS. These findings support the implementation of early, targeted intervention programs focused on improving physical function and managing weight to enhance quality of life and long-term health outcomes.

## Figures and Tables

**Table 1:** Comparison of body composition parameters between children with DS and TD children.

Variable	TD Children (M ± SD)	Children with DS (M ± SD)	*p*-Value	Mean Difference (95% CI)
Age	8.90 ± 1.14	9.73 ± 1.42	0.043 *	−0.82 (−1.62, −0.03)
Weight (kg)	25.52 ± 4.69	38.17 ± 12.17	0.001 *	−12.65 (−18.39, −6.91)
Height (cm)	125.43 ± 6.01	127.50 ± 10.17	0.424	−2.07 (−7.25, 3.11)
Body Fat Mass (kg)	3.48 ± 1.72	10.23 ± 5.62	0.001 *	−6.75 (−9.34, −4.17)
Soft Lean Mass (kg)	20.52 ± 3.84	25.75 ± 6.42	0.004 *	−5.23 (−8.51, −1.95)
Basal Metabolic Rate	1010.38 ± 73.92	1070.68 ± 92.6	0.031 *	−60.30 (−112.06, −8.54)
Lean Body Mass (kg)	22.05 ± 4.05	27.8 ± 7.19	0.006 *	−5.76 (−9.37, −2.14)
Total Body Water (L)	15.86 ± 2.95	20.03 ± 5.17	0.001 *	−4.17 (−6.78, −1.56)
Percentage of Body Fat (%)	14.05 ± 5.4	25.52 ± 6.33	0.001 *	−11.47 (−15.10, −7.84)
Body Mass Index (kg/m^2^)	16.1 ± 1.62	23.19 ± 4.45	0.001 *	−7.08 (−9.164, −5.00)
Fatness	26.8 ± 7.35	4.98 ± 23.72	0.001 *	−31.78 (−42.71, −20.86)
Body Fat Mass Arm Right	0.21 ± 0.11	0.66 ± 0.37	0.001 *	−0.44 (−0.61, −0.27)
Body Fat Mass Arm Left	0.22 ± 0.11	0.65 ± 0.35	0.001 *	−0.43 (−0.56, −0.27)
Body Fat Mass Trunk	1.82 ± 0.86	5.27 ± 2.87	0.001 *	−3.45 (−4.77, −2.13)
Body Fat Mass Leg Right	0.63 ± 0.3	1.84 ± 1.01	0.001 *	−1.21 (−1.67, −0.74)
Body Fat Mass Leg Left	0.62 ± 0.3	1.83 ± 1.02	0.001 *	−1.21 (−1.67, −0.74)
Soft Lean Mass Arm Right	1.38 ± 0.33	1.66 ± 0.47	0.025 *	−0.28 (−0.53, −0.02)
Soft Lean Mass Arm Left	1.33 ± 0.26	1.66 ± 0.48	0.013 *	−0.33 (−0.57, −0.09)
Soft Lean Mass Trunk	10.65 ± 2.02	13.11 ± 3.16	0.007 *	−2.46 (−4.13, −0.79)
Soft Lean Mass Leg Right	3.63 ± 0.72	4.6 ± 1.27	0.009 *	−0.98 (−1.61, −0.34)
Soft Lean Mass Leg Left	3.59 ± 0.68	4.61 ± 1.22	0.003 *	−1.02 (−1.64, −0.41)

TD: Typically developing children; DS: children with Down syndrome; CI: confidence interval; * statistically significant at 0.05.

**Table 2:** Comparison of postural stability characteristics between children with DS and TD children.

Variable	TD Children (M ± SD)	Children with DS (M ± SD)	*p*-Value	Mean Difference (95% CI)
Sitting to Standing	4	3.59 ± 0.5	0.001 *	0.41 (0.19, 0.63)
Standing to Sitting	4	3.59 ± 0.67	0.005 *	0.41 (0.12, 0.70)
Transfers	4	3.82 ± 0.39	0.043 *	0.18 (0.01, 0.36)
Standing Unsupported	4	4	-	-
Sitting Unsupported	4	4	-	-
Standing with Eyes Closed	3.95 ± 0.22	3.55 ± 0.51	0.002 *	0.41 (0.16, 0.65)
Standing with Feet Together	4	3.27 ± 0.83	0.001 *	0.73 (0.36, 1.09)
Standing with One Foot in Front	3.95 ± 0.22	2.18 ± 0.66	0.001 *	1.77 (1.46, 2.08)
Standing on One Foot	4	2.32 ± 0.89	0.001 *	1.68 (1.29, 2.08)
Turning 360 Degrees	4	3.27 ± 0.7	0.001 *	0.73 (0.42, 1.04)
Turning to Look Behind	4	3.27 ± 0.63	0.001 *	0.73 (0.45, 1.01)
Retrieving Object from Floor	4	3.64 ± 0.49	0.002 *	0.36 (0.15, 0.58)
Placing Alternate Foot on Stool	4	2.91 ± 0.68	0.001 *	1.09 (0.79, 1.39)
Reaching Forward with Outstretched Arm	3.86 ± 0.36	2.27 ± 0.55	0.001 *	1.58 (1.30, 1.87)
TBS	55.71 ± 0.9	45.77 ± 5.62	0.001 *	9.94 (7.43, 12.45)

TBS: Total balance score; TD: typically developing children; DS: children with Down syndrome; CI: confidence interval; * statistically significant at 0.05.

**Table 3:** Comparison of hand grip strength, physical activity, and aerobic endurance between children with DS and TD children.

Variable	TD Children (M ± SD)	Children with DS (M ± SD)	*p*-Value	Mean Difference (95% CI)
Hand Grip Strength (Dominant)	16.33 ± 3.61	13.73 ± 3.52	0.41	2.61 (0.41, 4.80)
Key Pinch Strength (Dominant)	3.9 ± 0.7	2.95 ± 0.84	0.001 *	0.95 (0.47, 1.43)
PA Score	4.05 ± 0.74	1.77 ± 0.75	0.001 *	2.28 (1.812, 2.74)
1 min Post-Exercise HR	91.43 ± 12.95	124.64 ± 11.16	0.001 *	−33.21 (−40.64, −25.77)

PA: Physical activity score; HR: heart rate; TD: typically developing children; DS: children with Down syndrome; CI: confidence interval; * statistically significant at 0.05.

**Table 4:** Correlation of body composition with demographic characteristics, postural stability, physical activity, muscle strength, and endurance in TD children (Pearson correlation).

Variable	Body Mass Index (BMI)	Lean Body Mass (LBM)	Body Fat Mass (MBF)
Age	r = 0.589 ** (*p* = 0.005)	r = 0.742 ** (*p* = 0.000)	r = 0.452 * (*p* = 0.039)
Gender	r = 0.245 (*p* = 0.285)	r = 0.496 * (*p* = 0.022)	r = −0.682 ** (*p* = 0.001)
Height	r = 0.583 ** (*p* = 0.006)	r = 0.808 ** (*p* < 0.001)	r = 0.467 * (*p* = 0.033)
TBS	r = 0.107 (*p* = 0.643)	r = 0.042 (*p* = 0.856)	r = 0.383 (*p* = 0.086)
PA Score	r = 0.485 * (*p* = 0.026)	r = 0.729 ** (*p* < 0.001)	r = 0.064 (*p* = 0.784)
Hand Grip Strength (Dominant)	r = 0.578 ** (*p* = 0.006)	r = 0.777 ** (*p* < 0.001)	r = 0.216 (*p* = 0.346)
Key Pinch Strength (Dominant)	r = 0.557 ** (*p* = 0.009)	r = 0.627 ** (*p* = 0.002)	r = −0.014 (*p* = 0.952)
1 min Post-Exercise HR	r = 0.123 (*p* = 0.596)	r = 0.011 (*p* = 0.962)	r = −0.077 (*p* = 0.741)
Fitness Category	r = 0.013 (*p* = 0.957)	r = 0.095 (*p* = 0.683)	r = 0.235 (*p* = 0.305)

TBS: Total balance score; PA: physical activity score; HR: heart rate; r: correlation coefficient; *p*: significance level; ** correlation is significant at 0.01; * correlation is significant at 0.05.

**Table 5:** Correlation of body composition with demographic characteristics, postural stability, physical activity, hand grip strength, and aerobic endurance in children with DS (Pearson correlation).

Variable	Body Mass Index (BMI)	Lean Body Mass (LBM)	Body Fat Mass (MBF)
Age	r = 0.661 ** (*p* = 0.001)	r = 0.892 ** (*p* < 0.001)	r = 0.895 ** (*p* < 0.001)
Gender	r = 0.007 (*p* = 0.974)	r = −0.185 (*p* = 0.411)	r = −0.430 * (*p* = 0.046)
Height	r = 0.577 ** (*p* = 0.005)	r = 0.866 ** (*p* < 0.001)	r = 0.895 ** (*p* < 0.001)
TBS	r = −0.697 ** (*p* < 0.001)	r = −0.827 ** (*p* < 0.001)	r = −0.684 ** (*p* < 0.001)
PA Score	r = −0.694 * (*p* < 0.001)	r = −0.536 * (*p* = 0.010)	r = −0.499 * (*p* = 0.018)
Hand Grip Strength (Dominant)	r = 0.688 ** (*p* < 0.001)	r = 0.891 ** (*p* < 0.001)	r = 0.810 ** (*p* < 0.001)
Key Pinch Strength (Dominant)	r = 0.436 * (*p* = 0.043)	r = 0.587 ** (*p* = 0.004)	r = 0.468 * (*p* = 0.028)
1 min Post-Exercise HR	r = 0.662 ** (*p* = 0.001)	r = 0.658 ** (*p* = 0.001)	r = 0.673 ** (*p* = 0.001)
Fitness Category	r = −0.714 ** (*p* < 0.001)	r = −0.476 * (*p* = 0.025)	r = −0.389 (*p* = 0.073)

TBS: Total balance score; PA: physical activity score; HR: heart rate; r: correlation coefficient; *p*: significance level; ** correlation is significant at 0.01; * correlation is significant at 0.05.

**Table 6:** Significant predictors for the body composition parameters in TD children (multiple linear regression).

	Body Mass Index (BMI)		Lean Body Mass (LBM)		Body Fat Mass (MBF)	
	β	Sig	β	Sig	β	Sig
Age	0.283	0.557	0.186	0.470	0.280	0.236
Gender			0.471	0.004	−0.727	*p* < 0.001
Height	0.380	0.361	0.768	0.003	0.280	0.234
Physical Activity Composite Score	−0.070	0.843	−0.163	0.421		
Hand Grip Strength_ Dominant	−0.241	0.665	−0.111	0.705		
Key Pinch Strength_ Dominant	0.477	0.123	0.127	0.442		
R Square (Sig)	0.480 (0.057)		0.868 (*p* < 0.001)		0.753 (*p* < 0.001)	

β: Standardized Coefficients Beta.

**Table 7:** Significant predictors for the body composition parameters in children with DS (multiple linear regression).

	Body Mass Index (BMI)		Lean Body Mass (LBM)		Body Fat Mass (MBF)	
	β	Sig	β	Sig	β	Sig
Age	−0.092	0.869	−0.371	0.303	0.068	0.894
Gender					−0.011	0.951
Height	0.291	0.481	0.585	0.039	0.594	0.095
Total Balance Score	−0.068	0.740	−0.226	0.101	0.107	0.556
PA Composite Score	−0.359	0.029	−0.117	0.230	−0.120	0.342
Hand Grip Strength_ Dominant	0.240	0.454	0.429	0.049	0.266	0.409
Key Pinch Strength_ Dominant	−0.010	0.951	0.071	0.505	−0.115	0.455
1 min Post-Exercise Heart Rate	−0.165	0.508	0.041	0.796	0.194	0.239
Fitness Category	−0.517	0.029	−0.113	0.413		
R Square (Sig)	0.825 (0.001)		0.929 (*p* < 0.001)		0.876 (*p* < 0.001)	

β: Standardized Coefficients Beta.

## Data Availability

The data presented in this study are available on request from the corresponding author.

## Abbreviations

The following abbreviations are used in this manuscript:

DS	Down Syndrome
TD	Typically Developing
PA	Physical Activity
MVPA	Moderate-to-Vigorous Physical Activity
CNS	Central Nervous System
ID	Intellectual Disability
ICC	Interclass Correlation
RHO	Spearman Rank-Order Correlation Coefficient
SD	Standard Deviation
SB	Sedentary Behavior
BMI	Body Mass Index
LBM	Lean Body Mass
MBF	Body Fat Mass
TBS	Total Balance Score
PAQ-C	Physical Activity Questionnaire for Children
PBS	Pediatric Balance Scale
BIA	Bioelectrical Impedance Analysis
3-MST	3-Minute Step Test
